# Comparison between Percutaneous Screw Fixation and Plate Fixation *via* Sinus Tarsi Approach for Calcaneal Fractures: An 8–10‐Year Follow‐up Study

**DOI:** 10.1111/os.12597

**Published:** 2019-12-18

**Authors:** Qi‐hao Weng, Gao‐le Dai, Qi‐ming Tu, Yang Liu, Vinesh Lutchooman, Jian‐jun Hong, Yang Yu

**Affiliations:** ^1^ Department of Orthopaedics The Second Affiliated Hospital and Yuying Children's Hospital of Wenzhou Medical University Wenzhou Zhejiang China; ^2^ The Second School of Medicine Wenzhou Medical University Wenzhou Zhejiang China

**Keywords:** Intra‐articular calcaneal fractures, Percutaneous reduction, Retrospective study, Sinus tarsi approach

## Abstract

**Objective:**

To assess the long‐term outcomes after percutaneous reduction (PR) and screw fixation versus plate fixation *via* the sinus tarsi approach (STA) for displaced intra‐articular calcaneal fractures (DIACF).

**Methods:**

This retrospective study included a total of 150 patients (June 2008–August 2011), comprising 85 men and 65 women (mean age, 38.4 years), who were assigned to the PR group or the STA group. The inclusion criteria were DIACF (>2 mm) including Sanders type II and III, closed fracture, unilateral fracture, no history of smoking or no smoking during hospitalization and 3 months after surgery, and follow‐up time not less than 8 years. The exclusion criteria were clear surgical contraindications (severe cardiovascular and cerebrovascular diseases), local or systemic infection symptoms, diagnosis with diabetes or lower extremity vascular disease, and Sanders type IV or open fractures. Outcomes were assessed by means of the American Orthopedic Foot and Ankle Society (AOFAS) hindfoot scores, radiographic images, and postoperative complications.

**Results:**

The mean follow‐up period was 8.7 years (range, 8.0–10.0 years). The AOFAS scores in the PR group during the follow‐up period were 54.2 ± 5.1, 85.8 ± 4.0, 88.1 ± 3.8, 87.9 ± 3.6, 87.8 ± 3.9, 86.9 ± 3.9, respectively, and in the STA group were 55.0 ± 5.6, 84.5 ± 5.2, 87.1 ± 3.8, 86.9 ± 3.8, 87.7 ± 3.3, and 87.6 ± 2.8, respectively. There was no significant difference in AOFAS scores, Bohler's angle, Gissane's angle, calcaneal length, and height between the two groups (*P* > 0.05). The good to excellent rate of the PR group (80.8%) was less than that of the STA group (91.7%) (*P* = 0.055). For Sanders III fractures, the good to excellent rate of the PR group (33.3%) was less than that of the STA group (76.9%) (*P* = 0.029). For calcaneal width recovery, the STA group performed better than the PR group (*P* < 0.05). The incidence of postoperative complications in the PR group (12.8%) was lower than that in the STA group (27.8%) (*P* = 0.026), of which the incidence of wound complications was 3.8% in the PR group and 13.9% in the STA group (*P* = 0.041). In addition, there was no significant difference in other postoperative complications such as sural nerve injury, peroneus longus and brevis muscle injury, calcaneal valgus symptoms, lateral impingement symptoms, and subtalar arthritis (*P* > 0.05).

**Conclusion:**

From the 8–10‐year follow‐up results of PR and STA as surgical procedures for the treatment of DIACF, it was found that there was no significant difference in the overall efficacy between them. STA was found to be superior to the PR in terms of the recovery of calcaneal width, providing more stable fixation for Sanders III fractures. PR was found to be more effective in reducing wound complications.

## Introduction

Approximately 75% of calcaneal fractures are intra‐articular fractures. Displaced intra‐articular fractures of the calcaneus are disabling injuries that occur mostly in young, active, physical laborers and, therefore, have a high socioeconomic impact. However, the treatment of displaced intra‐articular calcaneal fractures (DIACF) remains controversial^1^, ^2^. Historically, most surgeons have preferred non‐surgical treatment because of the unpredictability of surgical results^3^. In the past 15–20 years, further in‐depth understanding has been gained of the mechanism of intra‐articular fractures of the calcaneus; coupled with the improvement in surgical techniques and implants, there has been a rekindled interest in surgical fixation of fractures^4^, ^5^, ^6^. Operative reduction and fixation is now routinely recommended (with some exceptions) for DIACF. Studies with a large cohort of patients have shown that anatomical reconstruction of the calcaneal shape and joint shape can result in higher functional scores^7^. As a standard treatment for DIACF, open reduction and internal fixation (ORIF) *via* the extensile lateral approach (ELA) has been widely accepted and established^5^, ^8^. Studies have demonstrated a decrease in the incidence of late consequences and the socioeconomic burden of these injuries when treated by ORIF^5^, ^6^, ^9^. However, this surgical approach still has shortcomings. Because the skin of the lateral calcaneal wall is thin and vulnerable to injury, wound complications remain a major concern^7^. These complications mainly include wound edge necrosis, dehiscence, hematoma, infection, and sural nerve injury^10^, ^11^, ^12^, ^13^. The reported rate of wound edge necrosis ranges from 2% to 11%, the reported rate of soft tissue infection ranges from 1.3% to 7%, and the reported cumulative rate of wound complications is up to 25%^7^, ^10^. Therefore, many authors have proposed closed reduction and minimally invasive internal fixation to reduce the impact of wound complications, including arthroscopically assisted fixation, percutaneous fixation, and minimal incision techniques *via* lateral, posterior, or medial approaches^14^, ^15^, ^16^, ^17^, ^18^. It is reported that these methods effectively reduce the incidence of wound complications^19^.

These methods have also been discussed previously. In 1938, C. W. Goff described and illustrated more than 40 different operative treatment methods for displaced calcaneal fractures, most of which included some form of percutaneous reduction (PR) and skeletal traction^20^. In 1934, the method of closed reduction with percutaneous pin leverage and subsequent plaster immobilization was introduced by the German surgeon Westhues^21^. The approach was later modified and popularized in the English‐speaking literature by Gissane and Essex‐Lopresti^22^. More recently, this method has been advocated for tongue‐type fractures with the posterior calcaneal facet to the subtalar joint being displaced as a whole^14^. There are many ways to treat intra‐articular calcaneal fractures with minimally invasive surgery, and most of them have been reported many times. The choice of surgical method in the clinical treatment process is worth considering and it is meaningful to compare the therapeutic effects between minimally invasive surgical procedures. The comparison was made based on whether the fracture was an anatomic reduction, the incidence of postoperative complications, and the recovery of postoperative foot function. Among the procedures, STA and percutaneous fixation have been discussed more. As one of the most popular and effective minimally invasive surgical methods, the sinus tarsi approach (STA) can not only fully expose the posterior facet joint and the anterolateral segment but also reduce the incidence of postoperative wound complications^17^, ^23^, ^24^. Cheng *et al*. (2016)^25^ reported on percutaneous screw fixation and calcium sulfate cement grafting for the treatment of intra‐articular fractures. Compared with the traditional L‐shaped ELA, this technique allows early weight‐bearing and effectively reduces the incidence of wound complications and the stiffness of subtalar joints. Feng *et al*. (2006)^26^ compared the efficacy of percutaneous cannulated screw fixation and calcium sulfate cement grafting and STA in the treatment of DIACF. The clinical efficacy of the two methods was similar, but the incidence of wound complications of the former was lower than that of the latter, and the latter was better than the former in restoring calcaneal width.

We retrospectively analyzed 150 patients with intra‐articular calcaneal fractures who underwent percutaneous screw fixation and STA for an average follow‐up of 8.7 years. The purpose of this study was: (i) to evaluate the long‐term therapeutic effects of each of the two surgical methods; (ii) to compare the long‐term foot function scores of two minimally invasive methods; (iii) to compare the incidence of postoperative complications of the two methods; (iv) to evaluate anatomical restoration of the calcaneus under the two methods with X‐ray or CT measurement; (v) to compare the experimental results with the published literature and further evaluate the reliability of the experimental results; and (vi) to further guide clinical treatment with the results of this long‐term follow‐up.

## Materials and Methods

### *Participant Demographics*

This study was a retrospective clinical trial. Patients who were admitted to our hospital due to DIACF from June 2009 to November 2013 were selected. Two different surgical procedures were randomly performed on these patients according to the patients' wishes and the classification of the fracture. Some patients underwent PR and screw fixation, and others were treated with plate fixation *via* STA. The study was approved by the Second Hospital of Wenzhou Medical University Research Ethics Committee.

#### *Inclusion Criteria*

Inclusion criteria: (i) DIACF (>2 mm) including Sanders types II and III; (ii) closed fracture; (iii) unilateral fracture; (iv) no history of smoking or no smoking during hospitalization and 3 months after surgery; and (v) follow‐up time is not less than 8 years.

#### *Exclusion Criteria*

Exclusion criteria: (i) Clear surgical contraindications (severe cardiovascular and cerebrovascular diseases); (ii) local or systemic infection symptoms; (iii) diagnosis of diabetes or lower extremity vascular disease; and (iv) Sanders type IV or open fracture patients.

A total of 150 patients met the inclusion criteria, with 78 patients in the first group and 72 patients in the second group. The two groups of patients were categorized in terms of age, sex, cause of injury, and Sanders classification etc. (Table 1). The average time between hospitalization due to injury and the start of surgery was 5 (range, 1 to 7) days.

**Table 1:** Comparison of preoperative characteristics and follow‐up outcomes between two groups

Demographic data	PR Group (78 cases)	STA Group (72 cases)	*P‐*value
Age (years, mean ± SD)	39.0 ± 10.9	37.7 ± 11.5	0.722
Sex			0.792
Male	45	40	
Female	33	32	
Etiology			0.938
Fall from a height	60	55	
Traffic accident	18	17	
Sanders type			0.448
Sanders II	60	59	
Sanders III	18	13	
Good to excellent results (cases [%])			
Sanders type II	57 (95.0)	56 (94.9)	1
Sanders type III	6 (33.3)	10 (76.9)	0.029
Total	63 (80.8)	66 (91.7)	0.055
AOFAS scores (mean ± SD)			
BO	54.2 ± 5.1	55.0 ± 5.6	0.743
AO	85.8 ± 4.0	84.5 ± 5.2	0.692
1 y	88.1 ± 3.8	87.1 ± 3.8	0.433
3 y	87.9 ± 3.6	86.9 ± 3.8	0.397
5 y	87.8 ± 3.9	87.7 ± 3.3	0.862
8 y	86.9 ± 3.9	87.6 ± 2.8	0.549
Complications (cases)			
Wound complications	3	10	0.041
Superficial infection	2	7	
Deep infection	1	2	
Wound necrosis	0	1	
Sural nerve injury	1	2	0.608
Peroneus longus and brevis muscle injury	1	2	0.608
Calcaneal valgus symptoms	1	3	0.351
Lateral impingement symptoms	3	2	1
Subtalar arthritis	1	1	1
Total (cases [%])	10 (12.8)	20 (27.8)	0.026

1 y, 1 year after operation; AO, after operation; BO, before operation; PR, percutaneous reduction; STA, sinus tarsi approach.

#### *Surgical Method*

All patients were placed in the lateral position with the affected side facing up. After routine disinfection and anesthesia, both groups were operated on by the same orthopaedists.

#### *Percutaneous Reduction Group*

**Figure 1:**
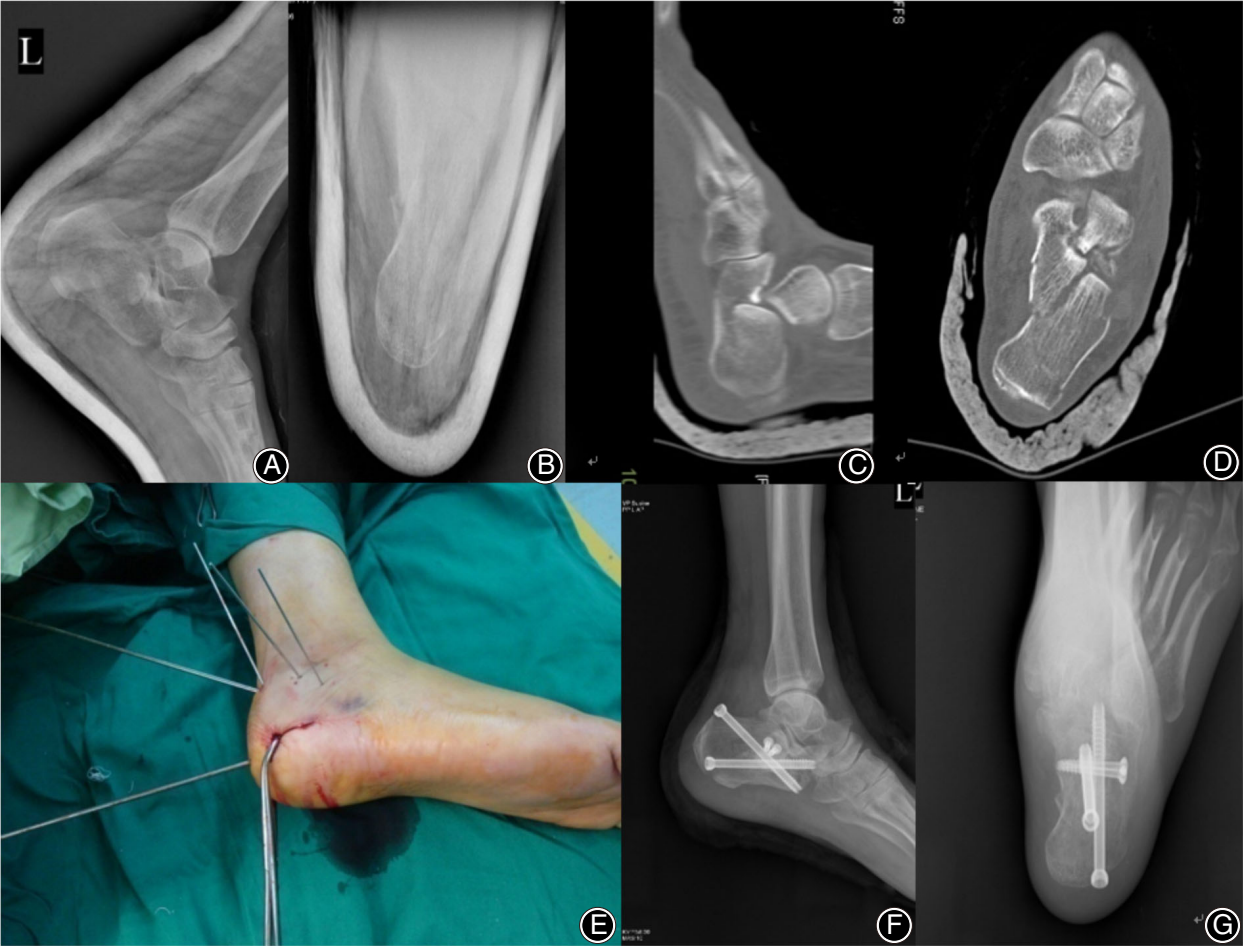
A 58‐year‐old female patient fell from height, resulting in an intra‐articular calcaneal fracture. (A) Preoperative lateral X‐ray and (B) preoperative axial X‐ray. CT image (C, D): A Sander type II calcaneal fracture with a subtalar articular surface collapse. (E) Intraoperative incision picture. (F) Postoperative lateral X‐ray shows a good reduction of calcaneal length, height, Bohler's angle, and Gissane's angle. (G) Postoperative axial X‐ray shows a good reduction of calcaneal width.

Under the guidance of C‐arm fluoroscopy, a 3.5‐mm Kirschner wire (K‐wire) was inserted percutaneously through the lateral aspect of the Achilles tendon at the upper posterior margin of the calcaneus. The K‐wire was then delivered slightly below the distal point of the posterior facet of the subtalar joint. The insertion angle was maintained at 15°–20° medially from the lateral margin of the foot and at 60°–70° from the plantar region. The tip of the K‐wire was stopped at approximately 1 cm below the posterior facet of the subtalar joint but outside the joint, touching the fracture bone block on the posterior calcaneal facet. Then the calcaneus and midfoot were bent towards the plantar side with the help of a K‐wire. In the last step, the posterior facet of the subtalar joint was moved closer to the sustentaculum tali by adjusting the rearfoot valgus. The effect of reduction was confirmed using the C‐arm X‐ray. A 1–2 1.5 mm K‐wire was then drilled medially from the posterior articular surface of the lateral bone block to transversely fix the fracture block of the sustentaculum tali. One K‐wire was drilled from the calcaneal tubercle towards the sustentaculum tali, and further towards the calcaneal axis to fix the primary and secondary fracture lines, respectively. Bohler's angle, Gissane's angle, and the length and height of the calcaneus were evaluated using the C‐arm X‐ray. After evaluation, 1–24.0‐mm cannulated screws were inserted from the calcaneal tubercle (Stryker) for transverse fixation of the bone block on the posterior facet of the subtalar joint (Fig. 1).

#### *Sinus Tarsi Approach Group*

**Figure 2:**
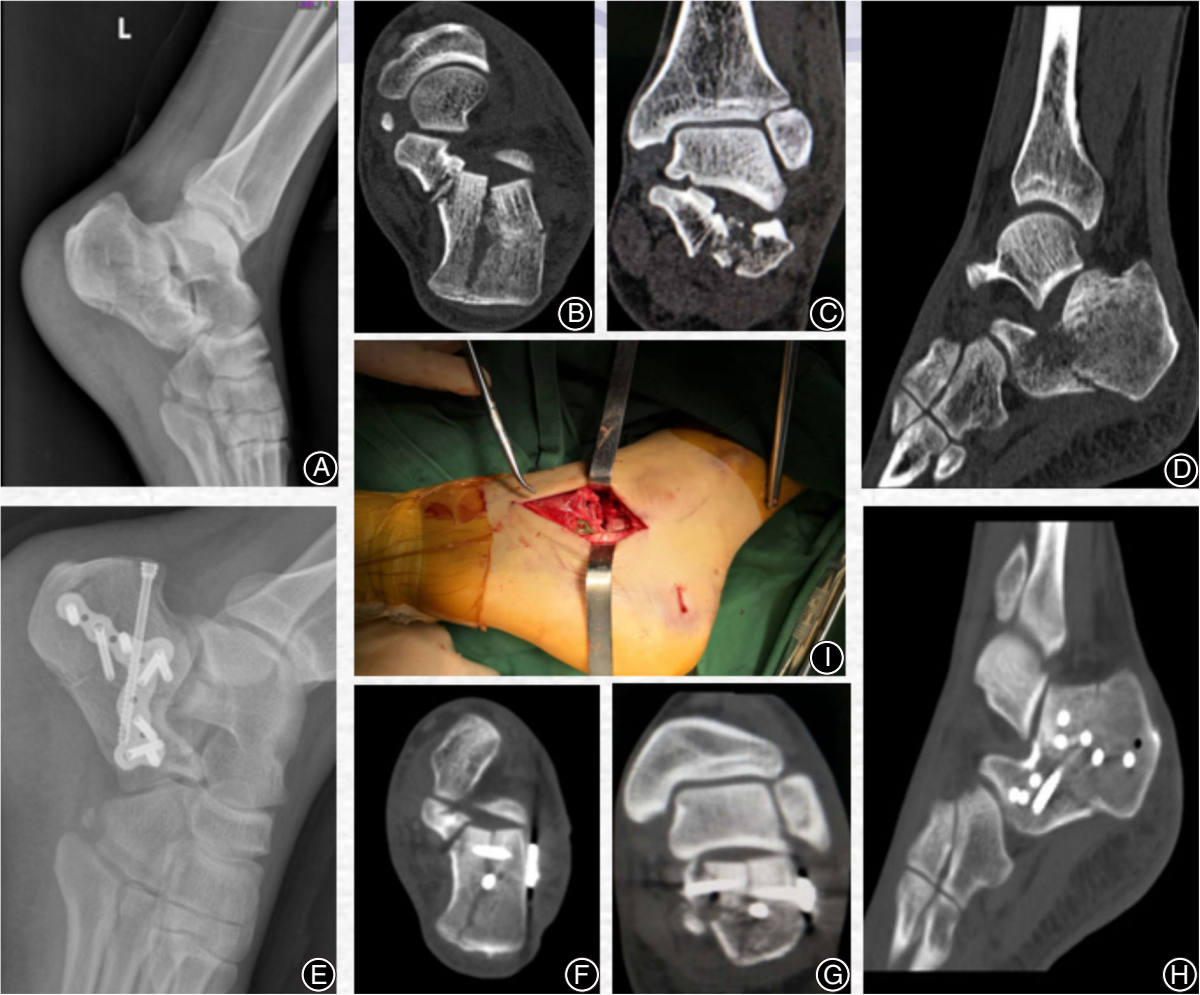
A 44‐year‐old female patient fell from height, resulting in an intra‐articular calcaneal fracture. (A) Preoperative lateral X‐ray. Preoperative CT scan shows (B–D): A Sander type III AB calcaneal fracture with a subtalar articular surface collapse and loss of Bohler's angle and Gissane's angle. (E) Postoperative lateral X‐ray. (F–H) Postoperative CT scan shows recovery of the subtalar articular surface, Bohler's angle, and Gissane's angle. (I) Intraoperative incision picture.

A 5–7‐cm incision parallel to the sole of the foot was made from the tip of the fibula to the lateral wall bone of the anterior process of the calcaneus. After exposing the visual field of the fracture and clearing the surface hematoma, the fracture fragments of the joint were repositioned by adjusting with a Steinmann pin. When a sufficient reduction by fluoroscopy under C‐arm X‐ray machine was confirmed, a 2–3 K‐wire was temporarily fixed. Then, squeezing of the lateral side of the calcaneus was performed by hand, supplemented with a poking reduction. Adjustment of the height of the calcaneus to expose the lateral calcaneal wall, the subtalar joint and the calcaneal body,and reposition the posterior articular surface of the subtalar joint. After the calcaneal length, height, and width were measured, Bohler's angle and Gissane's angle were determined using a C‐arm X‐ray machine. The plate was inserted through the incision. Following the plate insertion, the screws were fixed through the incision (Fig. 2).

### *Postoperative Treatment and Follow‐up*

There was no need for plaster cast fixation after surgery. Partial weight‐bearing of the forefoot was initiated 2 to 4 weeks after surgery. Partial weight‐bearing of the rear foot and walking exercises with crutches were initiated 6 to 8 weeks after surgery. After 12 weeks of surgery, fracture healing was evaluated by X‐ray image, after which the patients were allowed to begin full weight‐bearing and walking exercises. All patients were followed up for an average of 8.7 (range, 8.0–10.0) years.

### *Clinical Outcome*

The AOFAS hindfoot scores^26^ and the postoperative wound‐related complications were recorded. Lateral and axial X‐ray or CT scans were performed 1 day after the operation to assess the reduction of the fracture. Physical examination and lateral and axial X‐rays of the injured foot were performed at each follow‐up evaluation. Bohler's angle, Gissane's angle, the height, the width, and the length of the calcaneus were measured by X‐ray images or CT scans at each follow‐up.

#### *American Orthopedic Foot and Ankle Society Hindfoot Scores*

The AOFAS hindfoot scores were used to assess the functional outcome during the follow up. The AOFAS hindfoot score system mainly includes nine aspects: pain, function, maximum walking distance (blocks), walking surfaces, gait abnormality, sagittal motion (flexion plus extension), hindfoot motion (inversion plus eversion), ankle‐hindfoot stability (anteroposterior, varus‐valgus), and alignment. The score standard had a maximum of 100 points (best possible outcome). A total score less than 50 is considered a poor score, 50–74 fair, 75–89 good, and 90–100 excellent.

#### *Bohler's Angle and Gissane's Angle*

Bohler's angle and Gissane's angle can be measured using plain lateral radiographs of the calcaneus. Bohler's angle is formed by a line drawn from the highest point of the tuberosity to the highest point of the posterior facet and a line drawn from the highest point of the anterior process to the highest point of the posterior facet of the calcaneus. Gissane's angle is formed by a line drawn along the posterior facet of the calcaneus and a line drawn from the anterior process to the sulcus calcaneus. A normal Bohler's angle is 25° to 40° and a normal Gissane's angle is 100° to 130°. When the posterior articular surface collapses due to calcaneal fracture, Bohler's angle will become smaller or disappear, and Gissane's angle will become larger.

### *Statistical Analysis*

Statistical analysis was conducted using the SPSS18.0 package (SPSS, Chicago, IL, USA). Continuous measurements with normal distribution were expressed as mean ± standard deviation. The categorical measurements were statistically analyzed using Pearson χ^2^‐test (*n* > 40 and *t* > 5), Yates' correction for continuity (*n* > 40 and 1 < *t* < 5) or Fisher's exact test (*n* < 40 or *t* < 1), and the independent samples *t*‐test was used to compare the continuous measurements when the measurements appeared to be approximately normally distributed. *P* < 0.05 was considered statistically significant, referring to a two‐sided probability.

## Results

### *Functional Outcome*

As shown in Table 1, there was no significant difference in the AOFAS score between the PR group and the STA group at 1, 3, 5, and 8 years of follow‐up (*P* > 0.05). According to the AOFAS hindfoot scores, 63 patients were assessed as good and excellent in the PR group (57 in Sanders II and 6 in Sanders III, respectively) and 66 patients were assessed as good and excellent in the STA group (56 in Sanders II and 10 in Sanders III, respectively). Overall, there was no significant difference between the two groups in the good to excellent rate (80.8% *vs* 91.7%, *P* = 0.055, Table 1). As for Sanders III, the good to excellent rate in the PR group was significantly lower than in the STA group (33.3% *vs* 76.9%, *P* = 0.029, Table 1).

### *Postoperative Complications*

As shown in Table 1, 13 patients (3 in the PR group and 10 in the STA group) had wound healing complications, including superficial infections (2 in the PR group, 7 in the STA group), deep infection (1 in the PR group, 2 in the STA group), and wound necrosis (1 case in the STA group). The incidence of wound complications was 3.8% in the PR group and 13.9% in the STA group (*P* = 0.041). There was 1 case of sural nerve injury that occurred in the PR group and 2 cases in the STA group (*P* = 0.608). Peroneus longus and peroneus brevis injury occurred in 1 case in the PR group and 2 cases in the STA group (*P* = 0.608), which were sutured immediately intraoperatively and fixed with plaster for 4 weeks postoperatively. During the follow up, 2 patients developed subtalar arthritis (1 case in the PR group and 1 case in the STA group, *P* = 1.00). Both patients underwent subtalar arthrodesis. There were 5 patients with lateral impingement symptoms (3 in the PR group and 2 in the STA group, *P* = 1.00) and 4 patients with calcaneal valgus symptoms (1 in the PR group and 3 in the STA group, *P* = 0.351). Overall, the incidence of complications was 12.8% in the PR group and 27.8% in the STA group (*P* = 0.026). Internal fixation devices were removed 1 year after surgery in both groups of patients.

### *Radiographic Evaluation*

**Figure 3:**
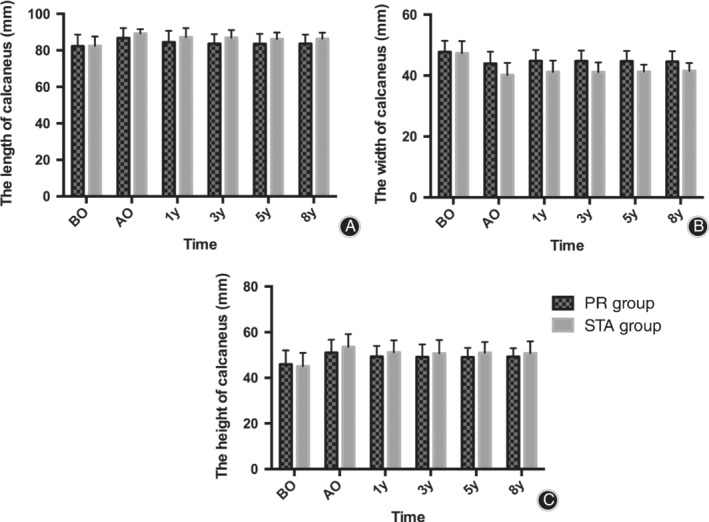
(A) Comparison of calcaneal length between the two groups preoperatively (*P* = 0.955), postoperatively (*P* = 0.205), and at 1 (*P* = 0.287), 3 (*P* = 0.136), 5 (*P* = 0.201), and 8 (*P* = 0.156) years of follow‐up. (B) Comparison of calcaneal width between the two groups preoperatively (*P* = 0.833), postoperatively (*P* = 0.047), and at 1 (*P* = 0.037), 3 (*P* = 0.025), 5 (*P* = 0.016), and 8 (*P* = 0.037) years of follow‐up. (C) Comparison of calcaneal height between the two groups preoperatively (*P* = 0.755), postoperatively (*P* = 0.348), and at 1 (*P* = 0.406), 3 (*P* = 0.528), 5 (*P* = 0.335), and 8 (*P* = 0.459) years of follow‐up. AO, after operation; BO, before operation.

**Figure 4:**
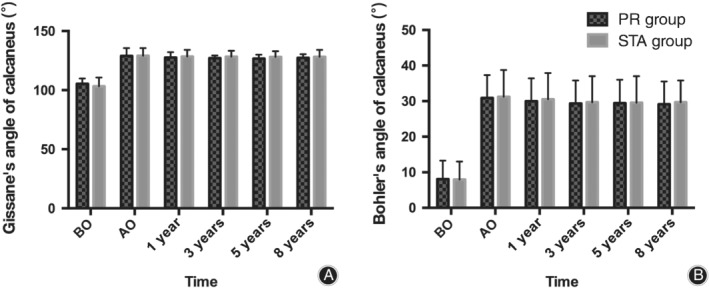
(A) Comparison of calcaneal Gissane's angle between the two groups preoperatively (*P* = 0.472), postoperatively (*P* = 0.968), and at 1 (*P* = 0.708), 3 (*P* = 0.473), 5 (*P* = 0.480), and 8 (*P* = 0.689) years of follow‐up. (B) Comparison of calcaneal Bohler's angle between the two groups preoperatively (*P* = 0.960), postoperatively (*P* = 0.923), and at 1 (*P* = 0.863), 3 (*P* = 0.920), 5 (*P* = 0.970), and 8 (*P* = 0.846) years of follow‐up. AO, after operation; BO, before operation.

As shown in Figs. 3 and 4, there was no significant difference in the calcaneal Bohler's angle, Gissane's angle, height, and length between the PR and STA groups during the preoperation, postoperation, and follow‐up period (*P* > 0.05). The calcaneal width was smaller in the STA group than in the PR group after the operation and during the follow‐up period (*P* < 0.05).

## Discussion

Calcaneal fractures account for 2.84% of all fractures and approximately 75% of them are DIACF^27^. The ELA is the most common approach for ORIF of DIACF and is supported by the currently available data^8^, ^28^, ^29^, ^30^. However, some studies have reported that this technique has a high incidence of postoperative complications, including wound edge necrosis, splitting, hematoma, or deep infection^31^, ^32^. To reduce wound complications, many operations for minimally invasive reduction and fixation of DIACF have been developed. In recent 30 years, percutaneous fixation, external fixation, arthroscopically assisted fixation and minimal incision techniques *via* medial, lateral, posterior or combined approaches have been described in published studies. The aim of our study was to compare the long‐term outcomes of DIACF treated by PR and screw fixation and plate fixation *via* STA.

### *Comparison of Clinical Outcomes between the Two Groups*

According to the AOFAS scores recorded during the follow‐up period, the average score in the PR group was 88.1, 87.9, 87.8, and 86.9 at 1, 3, 5, and 8 years after the operation, respectively. In the STA group, the average score was 87.1, 86.9, 87.7, and 87.6 at 1, 3, 5, and 8 years after the operation, respectively. The overall excellent rate between the two groups was 84.6% for PR and 90.3% for STA. There was no significant difference between the two groups, which indicated that the curative effect of the two surgical methods was similar. For the Sanders type II calcaneal fracture, the good to excellent rate of the PR group was 95.0% and the STA group was 94.9%. For the Sanders type III calcaneal fractures, the excellent rate of the PR group was 33.3%, which was less than 76.9% of the STA group, indicating that STA was more effective. In addition, the Sanders IIIAB outcomes were satisfactory while the results of the Sanders IIIAC and IIIBC were not satisfactory. PR and screw internal fixation for comminuted calcaneal fractures may increase the risk of insufficient reduction of the articular surface, resulting in low foot function scores^26^.

### *Comparison and Significance of Radiological Images*

The imaging results showed that the calcaneal width of the PR group was larger than that of the STA group postoperation and during follow‐up. Studies have shown that widening of the calcaneus is the main cause of lateral impingement syndrome^33^, ^34^. In this study, as for the lateral impact of the calcaneus, there were 3 cases in the PR group and 2 cases in the STA group. The difference between the two groups was not statistically significant. Bohler's angle and Gissane's angle are crucial for the anatomical reduction of the subtalar joint and restoration of normal width and height of the calcaneus, which affect the final treatment outcome^35^. As shown in Fig. 4, there were no statistically significant differences in Bohler's angle and Gissane's angle between the two groups during follow‐up.

### *Postoperative Complications*

In terms of the overall postoperative complication rate, the PR group had a significantly lower rate than the STA group, mainly for wound healing complications. Furthermore, the PR group had a lower risk of peroneus longus and brevis muscle injury, sural nerve injury, calcaneal valgus symptoms, and subtalar arthritis compared to the STA group, although the difference was not statistically significant. However, the lateral impingement symptoms were more profound in the PR group based on the experimental results. In Feng *et al*. (2016)^26^, the overall postoperative complication rates of the two surgical methods (percutaneous cannulated screw fixation *vs* calcium sulfate cement grafting and sinus tarsi approach) were 7.1% *vs* 28.9% and the difference was statistically significant. These results are similar to those of our study.

### *Previous Studies Involving the Two Surgical Approaches*

There have been many follow‐up studies on percutaneous fixation for calcaneal fractures. A 35‐month follow‐up study by Schepers *et al*.^36^ showed that the average score of AOFAS was 83.0 points and the incidence of infection complications was 15%. Rammelt *et al*.^16^ reported a study with an average follow‐up time of 29 months that showed an average AOFAS score of 92.1 points and no postoperative complications related to surgery were seen. A study by Tomesen *et al*.^37^ shows that at a mean follow‐up time of 66 months, the mean AOFAS score was 84.0 points and the postoperative infection rate of the wound was 13%. Follow‐up studies on the STA were also always reported. A retrospective study of Ebraheim *et al*.^38^ with an average follow‐up time of 29 months showed that the average score of AOFAS was 77.6 points and the rate of postoperative infection was 8.5%. A 31‐month follow‐up study by Weber *et al*.^39^ showed that the average score of AOFAS was 87.2 points and there were no wound complications. Zhang *et al*.^18^ show that at a mean follow‐up time of 27 months, the mean AOFAS scores was 88.8 points and the incidence of infection complications was 12.5%.

### *A Failed Case in the Study*

**Figure 5:**
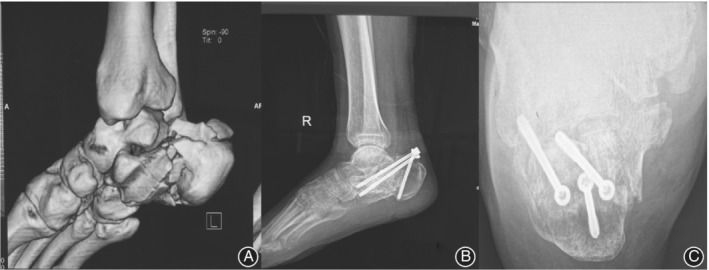
A 45‐year‐old female patient suffered from a Sander type III calcaneal fracture. (A) Preoperative three‐dimensional CT images (B, C). Postoperative X‐ray images showed that the reduction of calcaneal length, height, and width were not ideal.

Not all the operations were completed perfectly. As shown in Fig. 5, there was a failed case in the PR group. The patient had a Sanders type III calcaneal fracture. The length, height, and width of the calcaneus had not been restored and the articular surface had not been restored satisfactorily. This shows that the choice of surgical methods and surgical techniques is very important.

### *Limitations of the Study*

A limitation of this experiment is that the sample size is small, which may cause errors. Further follow‐up studies with large samples are needed. The data obtained in this study mainly depend on the records of follow‐up cases. Errors in the records of follow‐up cases or in the expression of patients will cause errors in this experiment. In addition, due to conditional limitations, the study was conducted only in our hospital and no multicenter randomized controlled trials were performed.

### *Conclusion*

This study involved a long‐term follow‐up comparing PR and screw fixation and plate fixation *via* STA for patients with DIACF. STA was found to be superior to PR in terms of the recovery of calcaneal width, providing more stable fixation for Sanders III fractures. PR was found to be more effective in reducing wound complications. The two techniques had similar AOFAS scores after the operation.
